# Safety of Vancomycin Use Through Midline Catheters for Outpatient Parenteral Antimicrobial Therapy

**DOI:** 10.1001/jamainternmed.2025.3110

**Published:** 2025-07-21

**Authors:** David Paje, Emily Walzl, Megan Heath, Elizabeth McLaughlin, Jennifer K. Horowitz, Caitlin Tatarcuk, Lakshmi Swaminathan, Scott Kaatz, Anurag N. Malani, Ashwin Gupta, Valerie M. Vaughn, Steven J. Bernstein, Scott A. Flanders, Vineet Chopra

**Affiliations:** 1 Division of Hospital Medicine, Department of Internal Medicine, Michigan Medicine, University of Michigan, Ann Arbor; 2 The Hospital Medicine Safety Consortium Coordinating Center, Ann Arbor, Michigan; 3 Medicine Service, VA Ann Arbor Healthcare System, Ann Arbor, Michigan; 4 Section of Hospital Medicine, Trinity Health Michigan, Ann Arbor; 5 Division of Hospital Medicine, Henry Ford Health, Detroit, Michigan; 6 Section of Infectious Diseases, Trinity Health Michigan, Ann Arbor; 7 Division of General Internal Medicine, Department of Internal Medicine, University of Utah, Salt Lake City; 8 Division of General Medicine, Department of Internal Medicine, Michigan Medicine, University of Michigan, Ann Arbor; 9 Center for Clinical Management Research, VA Ann Arbor Healthcare System, Ann Arbor, Michigan; 10 Department of Medicine, University of Colorado Anschutz Medical Campus, Aurora

## Abstract

This cohort study examines the association between vancomycin use through midline catheters and device-related complications among patients receiving outpatient parenteral antimicrobial therapy.

Midline catheters are alternatives to peripherally inserted central catheters for outpatient parenteral antimicrobial therapy (OPAT).^1^ Current guidelines recommend against using midline catheters for extended treatment with vancomycin due to its irritant and vesicant properties.^2,3^ However, evidence supporting this recommendation is limited, and whether such practice is prevalent or harmful remains unknown. We analyzed data from the Michigan Hospital Medicine Safety Consortium (HMS) to assess device-related complications among hospitalized patients receiving OPAT with and without vancomycin therapy.

## Methods

We used data from hospitalized patients who received midline catheters between January 2017 and August 2024. We excluded patients who received their device in critical care settings and included patients who received antimicrobials through a midline catheter after hospital discharge.^1^ Patients were followed up until device removal, death, or 30 days from device insertion. This study was classified as not regulated by University of Michigan’s Institutional Review Board; informed consent was waived because all data were deidentified. This study followed the STROBE reporting guideline.

The primary outcome was a major device complication, including catheter-related bloodstream infection (CRBSI) and catheter-related venous thromboembolism (CR-VTE). Secondary outcomes included device failure, defined as premature removal of midline catheter due to any complication as described.^1^ We assessed associations between receipt of vancomycin and outcomes using Fine-Gray hazards models, accounting for dwell time and risk of competing event of device removal from other device complications, adjusting for patient and device characteristics.^4^ Adjusted hazard ratios (aHRs) with 95% CIs were calculated using SAS version 9.4 (SAS Institute). All tests were 2-tailed, and *P* < .05 was considered statistically significant (eMethods in Supplement 1).

## Results

Among 3317 patients prescribed OPAT through midline catheters, the median (IQR) age was 67.5 (56.1-77.6) years; 1678 (51.5) were male and 1639 (49.4%) were female. A total of 597 (18.0%) received vancomycin, whereas 2720 (82.0%) did not. OPAT duration (median [IQR], 11 [7-17] vs 11 [7-16] days; *P* = .33), catheter dwell time (median [IQR], 12.0 [8.0-19.0] vs 12.0 [8.0-17.0] days; *P* = .14), and consultation with infectious diseases (ID) specialists (84.9% vs 82.8%; *P* = .22) were similar between both groups.

Major device complications occurred in 49 (1.5%) of 3317 patients in the overall cohort, including 27 (4.5%) of 597 vancomycin recipients vs 22 (0.8%) of 2720 nonrecipients (*P* < .001). Vancomycin recipients had higher rates of CRBSI (2.5% vs 0.3%; *P* < .001) and CR-VTE (2.0% vs 0.6%; *P* < .001) than nonrecipients. Device failure was more prevalent among vancomycin recipients than nonrecipients (16.9% vs 9.6%; *P* < .001). In adjusted analyses, vancomycin administration through a midline catheter was associated with a higher hazard of major device complications (aHR, 4.82; 95% CI, 2.64-8.83) and device failure (aHR, 1.75; 95% CI, 1.36-2.25; Table; Figure). Vancomycin was also associated with higher risks of CRBSI (aHR, 8.00; 95% CI, 2.96-21.63) and CR-VTE (aHR, 3.30; 95% CI, 1.52-7.16). Results were robust to sensitivity analyses, including patients discharged home (vs facility) and patients who received vancomycin monotherapy.

**Table.** Incidence Density and Hazards of Device Complications in OPAT Through Midline Catheters, by Receipt of Vancomycin

Outcomes	Total No. (No. per 1000 catheter-days)			Adjusted HR (95% CI)^a^	*P* value
	Total (N = 3317)	Vancomycin (n = 597)	No vancomycin (n = 2720)		
Any major complication	49 (1.10)	27 (3.27)	22 (0.61)	4.82 (2.64-8.83)	<.001
Catheter-related BSI	22 (0.49)	15 (1.82)	7 (0.19)	8.00 (2.96-21.63)	<.001
Catheter-related VTE	27 (0.61)	12 (1.45)	15 (0.41)	3.30 (1.52-7.16)	.003
Upper extremity DVT	20 (0.45)	7 (0.85)	13 (0.36)	1.51 (0.64-3.58)	.35
Pulmonary embolism	7 (0.16)	5 (0.61)	2 (0.06)	NA	NA
Any minor complication	379 (8.50)	92 (11.14)	287 (7.90)	1.47 (1.14-1.90)	.003
Catheter dislodgement	123 (2.76)	26 (3.15)	97 (2.67)	1.14 (0.69-1.86)	.61
Catheter occlusion	89 (2.00)	22 (2.66)	67 (1.84)	1.69 (0.94-3.02)	.08
Catheter tip migration	40 (0.90)	11 (1.33)	29 (0.80)	1.74 (0.83-3.69)	.15
Infiltration	23 (0.52)	10 (1.21)	13 (0.36)	3.92 (1.70-9.03)	.001
Superficial thrombosis	30 (0.67)	6 (0.73)	24 (0.66)	1.48 (0.58-3.77)	.42
Exit site concerns	146 (3.28)	40 (4.84)	106 (2.92)	1.81 (1.22-2.70)	.003
Any major or minor complication	410 (9.20)	110 (13.32)	300 (8.26)	1.67 (1.32-2.11)	<.001
Device failure	362 (8.12)	101 (12.23)	261 (7.19)	1.75 (1.36-2.25)	<.001

Abbreviations: BSI, bloodstream infection; DVT, deep vein thrombosis; HR, hazard ratio; OPAT, outpatient parenteral antimicrobial therapy; NA, not applicable; VTE, venous thromboembolism.

^a^ HR was calculated using a Fine-Gray regression model adjusted for competing event of device removal due to other device complications, dwell time, age, sex, Charlson Comorbidity Index score, history of VTE, history of central line-associated BSI, active malignancy, receipt of anticoagulants, presence of another central vein catheter, cut catheter tip, number of catheter lumens, and catheter size, with random effects for each hospital.

**Figure.**
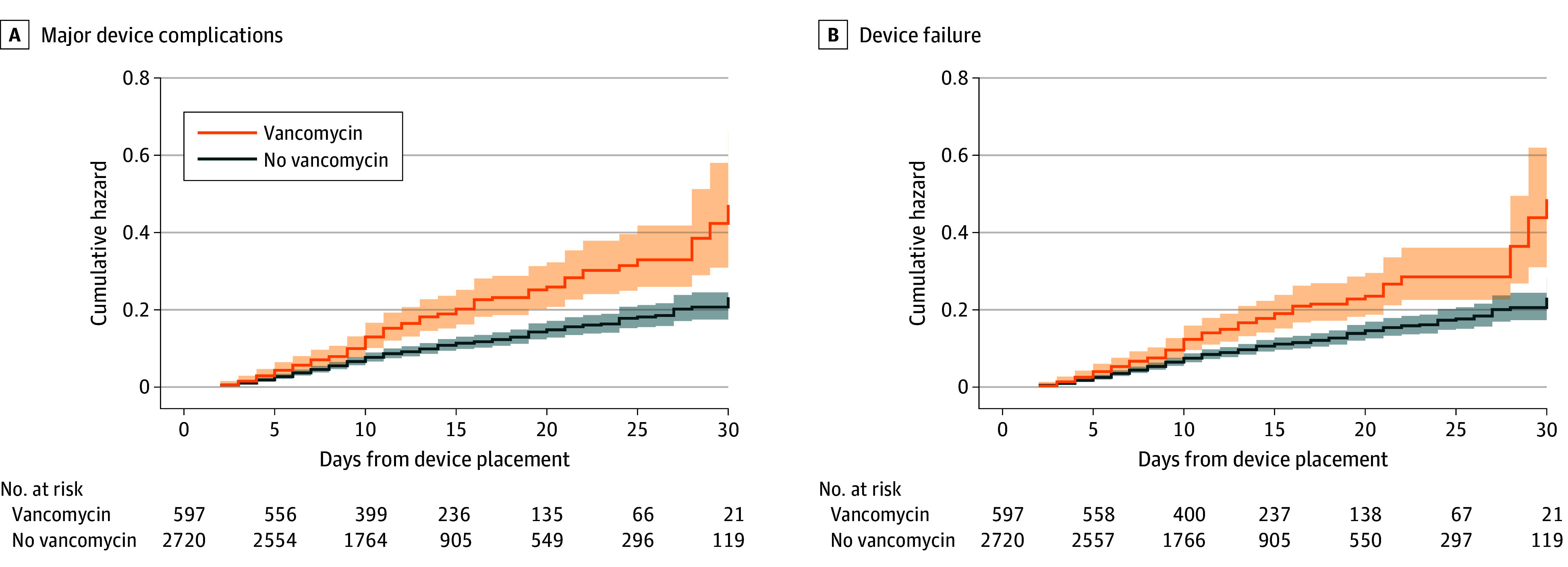
Cumulative Hazard Curves of Patients Receiving Outpatient Parenteral Antimicrobial Therapy (OPAT) Through Midline Catheters, by Receipt of Vancomycin Shaded areas represent 95% CIs.

## Discussion

In this multicenter study of patients discharged with midline catheters for OPAT, vancomycin infusion was associated with higher risk of major device complications and premature catheter removal. Major device complications were 5 times more likely among vancomycin recipients than nonrecipients, arising from an 8-fold greater risk of CRBSI and 3-fold greater risk of CR-VTE. Our findings support current recommendations against using midline catheters for vancomycin infusion.^2^ However, nearly 1 in 5 midline catheters in our cohort were used for vancomycin-based OPAT, and 4 of 5 recipients were seen by an ID specialist before device placement. Study limitations included its observational design, secondary use of medical records, and lack of information regarding events after midline catheter removal.

These findings highlight opportunities for practice improvement, especially given widespread availability of intravenous and oral alternatives to vancomycin.^5,6^ They also underscore the importance of infusate compatibility in ensuring the safety and reliability of midline catheters. Collaboration among vascular access teams, ID specialists, pharmacists, and ambulatory infusion nurses is necessary to optimize practice and prevent harm from using midline catheters for vancomycin-based OPAT.

## Notes

Supplement 1.
eMethods.

Supplement 2.
Data Sharing Statement
