# Supplementary material for: Safety of Vancomycin Use Through Midline Catheters for Outpatient Parenteral Antimicrobial Therapy
Source: JAMA Intern Med. 2025 Jul 21;185(9):1162–4. doi: 10.1001/jamainternmed.2025.3110 (PMC12281390; doi:10.1001/jamainternmed.2025.3110)
Supplement: Supplement 1. — eMethods. [file jamainternmed-e253110-s001.pdf]

Supplemental Online Content

Paje D, Walzl E, Heath M, et al. Safety of vancomycin use through midline catheters for outpatient parenteral antimicrobial therapy. *JAMA Intern Med*. Published online July 21, 2025. doi:10.1001/jamainternmed.2025.3110

eMethods.

This supplemental material has been provided by the authors to give readers additional information about their work.

© 2025 Paje D et al. JAMA Internal Medicine.

eMethods.

Study Setting

The HMS consortium is a collaborative quality initiative that aims to improve the quality of care for hospitalized medical patients. It includes 69 (75%) of 92 nonfederal, noncritical access hospitals across the State of Michigan, and supported by Blue Cross Blue Shield of Michigan and Blue Care Network as part of the BCBSM Value Partnerships program. The design and protocols of HMS have been previously described.[1, 2]

At each participating hospital, an HMS consortium-trained and -supported abstractor collected patient-level data from the electronic health records on a sample of midline catheters placed in medical and critically ill patients following a standard protocol and data collection tool. Since HMS is focused on hospitalized adults, patients that were (a) under 18 years, (b) pregnant, (c) admitted for palliative care, (d) admitted to a nonmedical service (e.g., surgery), or (e) admitted under observation status, were excluded. At every 14-day cycle, the first six patients meeting selection criteria were enrolled and data were captured from the time of midline catheter placement. Patients were followed until midline catheter removal, death, or 30 days from midline catheter insertion, whichever came first. Data were manually collected through review of medical records, including clinical documentation and results of imaging studies and laboratory/microbiological testing, and through 30-day follow-up telephone interviews with patients. To assure data quality and integrity, the HMS coordinating center at University of Michigan performed annual audits at each participating hospital.

© 2025 Paje D et al. JAMA Internal Medicine.

This study was classified as “not regulated” by the institutional board at the University of Michigan, and informed consent was not required as all data were deidentified (HUM00078730). We followed the Strengthening the Reporting of Observational Studies in Epidemiology (STROBE) reporting guidelines.

Patients

We used data from patients who had midline catheters placed between January 2017 and August 2024 while admitted to a general medical unit. Consistent with recent work and to ensure a homogenous cohort that represents an OPAT population, we excluded patients who received midline catheters while in a critical care unit setting. Likewise, we only included patients who received antimicrobials through their midline catheter that began while admitted to the hospital and continued beyond hospital discharge.[3]

Covariates

Data on patient characteristics at the time of midline catheter insertion were abstracted from electronic medical records (EMR), including demographic data, detailed clinical history, medications, level of care, and laboratory test results. Information on discharge location was obtained from review of EMR or telephone interview with patients. Charlson comorbidity index was used to quantify comorbidity burden. Clinician (e.g., type of professional performing the midline catheter insertion and number of insertion attempts) and device (e.g., number of catheter lumens and catheter size) information were abstracted from the midline catheter insertion note. Hospital characteristics (e.g., number of hospital beds and teaching status) were obtained from

© 2025 Paje D et al. JAMA Internal Medicine.

publicly reported data and information provided to HMS by participating hospitals.

Outcomes

The primary outcome was a major device complication, which included catheter-related bloodstream infection (CRBSI) and catheter-related venous thromboembolism (CR-VTE). CRBSI was defined in accordance with Centers for Disease Control criteria as laboratory-confirmed bloodstream infection (BSI) with midline catheter in situ and no other identified source of BSI, or if a physician documented line sepsis or catheter-related bacteremia/fungemia, or if the midline catheter was removed due to suspected CRBSI.[4] CR-VTE was defined as symptomatic, image-confirmed, upper extremity deep vein thrombosis or symptomatic image-confirmed pulmonary embolism.

Secondary outcomes were classified as minor device complications (i.e., catheter dislodgement, occlusion, tip migration, infiltration, superficial thrombophlebitis, or exit site concerns [including leaking, discharge, or infection]) and device failure, which was defined as removal of midline catheter due to any complication. Device failure is a relevant outcome metric because device complications are competing events that may lead to premature device removal (e.g., occlusion) and thus affect primary outcomes (e.g., CRBSI). Device failure also serves as a surrogate to understand the net effect of device complications on patient experience and device efficacy in delivering OPAT. As midline catheters may lose blood return after a few days while still maintaining the ability to flush, catheter occlusion was defined as the inability to flush or infuse, or an intervention was performed to declot or “clear” the catheter (e.g., instillation of thrombolytic agent). Published and validated definitions were used to capture all major

© 2025 Paje D et al. JAMA Internal Medicine.

and minor midline catheter complications.[4, 5]

Statistical Analysis

Cohort information, including patient, clinician and device characteristics were summarized using descriptive statistics. Patients on OPAT who received vancomycin were compared to those who did not receive vancomycin using Chi-square test for categorical variables and Wilcoxon test for continuous variables. Associations between receipt of vancomycin for OPAT and outcomes were assessed using Fine-Gray hazards models, accounting for device dwell time and risk for competing event of device removal due to other device complications.[6] Models were adjusted for patient age, sex, comorbidities (using the Charlson index), history of VTE, history of CLABSI, active malignant neoplasm, receipt of anticoagulants, presence of a central vein catheter, cut catheter tip, number of catheter lumens, and catheter size, with random effects for hospital-level variation. To account for practice variation and potential confounding of outcomes associated with stay in post-acute care facilities, we performed sensitivity analyses limited to patients whose discharge location was home. Additionally, we performed sensitivity analyses limited to vancomycin monotherapy recipients vs. patients that received vancomycin and other antimicrobials following discharge.

Results were expressed as adjusted hazard ratios (aHRs) with 95% confidence intervals (CIs). All tests were 2-tailed, and $P<.05$ was considered statistically significant. We performed all statistical analyses in SAS version 9.4 (SAS Institute, USA).

© 2025 Paje D et al. JAMA Internal Medicine.

eReferences

1. Paje, D., et al., *Use of Peripherally Inserted Central Catheters in Patients With Advanced Chronic Kidney Disease: A Prospective Cohort Study.* Ann Intern Med, 2019. **171**(1): p. 10-18.
2. Paje, D., et al., *Midline catheters in patients with advanced chronic kidney disease.* J Hosp Med, 2023. **18**(11): p. 969-977.
3. Paje, D., et al., *Midline vs Peripherally Inserted Central Catheter for Outpatient Parenteral Antimicrobial Therapy.* JAMA Intern Med, 2025. **185**(1): p. 83-91.
4. *Bloodstream Infection Event (Central Line-Associated Bloodstream Infection and Non-central Line Associated Bloodstream INfection)*. National Healthcare Safety Network 2024 [cited 2024 6/10/2024]; Available from: https://www.cdc.gov/nhsn/pdfs/pscmanual/4psc_clabscurrent.pdf.
5. Nickel, B., et al., *Infusion Therapy Standards of Practice, 9th Edition.* J Infus Nurs, 2024. **47**(1S Suppl 1): p. S1-S285.
6. Fine, J.P. and R.J. Gray, *A Proportional Hazards Model for the Subdistribution of a Competing Risk.* Journal of the American Statistical Association, 1999. **94**(446): p. 496-509.

© 2025 Paje D et al. JAMA Internal Medicine.
