# Supplementary material for: Safety of Vancomycin Use Through Midline Catheters for Outpatient Parenteral Antimicrobial Therapy
Source: JAMA Intern Med. 2025 Jul 21;185(9):1162–4. doi: 10.1001/jamainternmed.2025.3110 (PMC12281390; doi:10.1001/jamainternmed.2025.3110)
Supplement: Supplement 2. — Data Sharing Statement [file jamainternmed-e253110-s002.pdf]

Data Sharing Statement

Paje. Safety of Vancomycin Use Through Midline Catheters for Outpatient Parenteral Antimicrobial Therapy. *JAMA Intern Med*. Published July 21, 2025. doi:10.1001/jamainternmed.2025.3110

Data
Data available: No

Additional Information
Explanation for why data not available: As our initiative is a collaborative of hospitals in Michigan focusing on improving the quality of care for hospitalized patients with PICCs and midline catheters, we utilized the existing infrastructure of the Michigan Hospital Medicine Safety (HMS) Consortium. The data use agreements among the participating hospitals prohibit us from sharing our registry data outside of the Coordinating Center, given hospital specific performance is directly identifiable. For further information, please contact the Institutional Review Borad at the University of Michigan (HUM 00078730) @ irbmed@umich.edu.
